# Paternal Aging Affects Behavior in *Pax6* Mutant Mice: A Gene/Environment Interaction in Understanding Neurodevelopmental Disorders

**DOI:** 10.1371/journal.pone.0166665

**Published:** 2016-11-17

**Authors:** Kaichi Yoshizaki, Tamio Furuse, Ryuichi Kimura, Valter Tucci, Hideki Kaneda, Shigeharu Wakana, Noriko Osumi

**Affiliations:** 1 Department of Developmental Neuroscience, Tohoku University Graduate School of Medicine, Sendai, Miyagi, Japan; 2 Technology and Development Team for Mouse Phenotype Analysis, The Japan Mouse Clinic, RIKEN BRC, Tsukuba, Ibaraki, Japan; 3 Department of Neuroscience and Brain Technologies. Istituto Italiano di Tecnologia, Genova, Italy; Tokyo Metropolitan Institute of Medical Science, JAPAN

## Abstract

Neurodevelopmental disorders such as autism spectrum disorder (ASD) and attention deficit and hyperactivity disorder (ADHD) have increased over the last few decades. These neurodevelopmental disorders are characterized by a complex etiology, which involves multiple genes and gene-environmental interactions. Various genes that control specific properties of neural development exert pivotal roles in the occurrence and severity of phenotypes associated with neurodevelopmental disorders. Moreover, paternal aging has been reported as one of the factors that contribute to the risk of ASD and ADHD. Here we report, for the first time, that paternal aging has profound effects on the onset of behavioral abnormalities in mice carrying a mutation of *Pax6*, a gene with neurodevelopmental regulatory functions. We adopted an *in vitro* fertilization approach to restrict the influence of additional factors. Comprehensive behavioral analyses were performed in *Sey/+* mice (i.e., *Pax6* mutant heterozygotes) born from *in vitro* fertilization of sperm taken from young or aged *Sey/+* fathers. No body weight changes were found in the four groups, i.e., *Sey/+* and wild type (WT) mice born to young or aged father. However, we found important differences in maternal separation-induced ultrasonic vocalizations of *Sey/+* mice born from young father and in the level of hyperactivity of *Sey/+* mice born from aged fathers in the open-field test, respectively, compared to WT littermates. Phenotypes of anxiety were observed in both genotypes born from aged fathers compared with those born from young fathers. No significant difference was found in social behavior and sensorimotor gating among the four groups. These results indicate that mice with a single genetic risk factor can develop different phenotypes depending on the paternal age. Our study advocates for serious considerations on the role of paternal aging in breeding strategies for animal studies.

## Introduction

Autism spectrum disorders (ASD) and attention deficit and hyperactivity disorder (ADHD) are the most frequent neurodevelopmental disorders that receive diagnosis early in childhood. These developmental conditions have progressively increased in recent year, causing a significant burden for the society [1, 2]. ASD exhibits complex symptoms, which include abnormalities in social interaction and repetitive behaviors. Moreover, ASD patients often exhibit motor deficits, sensorimotor dysfunction, epilepsy and anxiety, and in some cases it has been reported the presence of ADHD symptoms [3–8]. Although several twin and family studies proved the existence of significant genetic contributions to the etiology of the ASD [9–12], the high heritability of specific traits did not facilitate the identification of the specific genetic causes of the disorder [13, 14]. However, recent genome-wide screening of candidate genes has indicated clustering of molecules involved in synaptic signaling and chromatin remodeling as plausible targets [15, 16].

*Pax6* is a highly conserved transcriptional factor among vertebrates and is crucial for brain development by regulating expression of many downstream genes in highly context-dependent manners [17–22]. It has been recently reported that Pax6 interacts with chromatin remodeling complexes such as BAF and CTCF [23–25]. Human *PAX6* gene was originally identified as a responsible gene for aniridia in the chromosome region 11p13 that is responsible for WAGR (Wilim’s tumor, Aniridia, Genitourinary malformations and mental Retardation) syndrome [26, 27]. Since then, several reports showed that mutations in *PAX6* gene are risk factors for ASD and related disorders [28–32]. Furthermore, several studies have shown structural brain abnormalities in people with mutations in *PAX6* gene [33–36]. We previously studied the behavior of spontaneous *Pax6* mutant heterozygous (*rSey*^*2*^/+) rats as a model of neurodevelopmental dysfunctionality and found deficits in ultrasonic vocalizations, social behavior, emotional behavior, sensorimotor gating and fear-conditioned memory [37]. Cortex-specific *Pax6* knockout mice (*Emx1-Cre*; *Pax6*^*fl/fl*^ mice) also showed deficiencies in sensorimotor information integration and both hippocampus-dependent short-term and neocortex-dependent long-term memory recalls [38]. In addition we recently reported decrease in volume of various brain regions in *rSey*^*2*^/+ rats from the MRI study [39]. Therefore, we hypothesized a role of *Pax6* in animal behavior and in neurodevelopmental abnormalities such as those characterizing ASD and related neurodevelopmental disorders.

Although ASD exhibits high heritability, various environmental factors are suggested to be potential confounders. In particular, we have investigated the role of paternal aging. Paternal aging has recently received a particular attention as an important factor that contributes to the etiopathogenesis of many psychiatric disorders, including ASD and ADHD [40–45]. The detrimental effects of paternal aging has been largely investigated in rodents; for example, offspring derived from aged father showed learning deficits, impaired social behavior, hyperactivity and anxiety traits [46–48]. However, the interplay between paternal aging and a genetic risk is still poorly understood.

In the present study, we conducted comprehensive behavioral analyses in *Sey/+* mice derived from young or aged father. To minimize the effects of additional factors, we adopted *in vitro* fertilization (IVF) to obtain offspring. We found that different behavioral phenotypes between *Sey/+* mice born from young or aged father; *Sey/+* mice born from young father showed deficits in ultrasonic vocalizations. In contrast, *Sey/+* mice born from aged father exhibited more hyperactivity and less immobility. Anxiety-related phenotypes were commonly observed in both genotypes born from aged father but not in young father. These results reinforce a notion that different phenotypes can occur in combination of a single mutation and paternal aging.

## Materials and Methods

### Ethical statement

All procedures described here were reviewed and approved by the Animal Experimentation Committee at RIKEN (No.10-013) and Ethic Committee for Animal Experiments of Tohoku University Graduate School of Medicine (#2013–390), and were performed in accordance with the National Institute of Health guidance for the care and use of laboratory animals. No animals exhibited symptoms indicative of severe illness/moribundity during the implementation period.

### Mouse production for behavioral analyses

To reproduce the progeny for the behavioral analyses, IVF was performed. *Sey/+* and wild type (WT) mice (C57BL6/JCrj background) were born from IVF using sperm collected from one young (3 month-old) *Sey/+* male mouse and one aged (12 month-old) *Sey/+* male mouse and eggs collected from female C57BL6/JCrj mice (3 to 4 week-old) that were purchased from Charles River Laboratories International, Inc. (Yokohama, Japan). Sperm were collected from the caudae epididymides of male mice, and allowed to diffuse in fertilization medium. After pre-incubation for approximately 1 hour to allow for capacitation, the sperm were used for insemination. Meanwhile, female mice were superovulated using intraperitoneal injections of PMSG and HCG (Serotropin and Gonatropin; ASKA Pharmaceutical Co., Tokyo, Japan) with an interval of 48 hours between injections. Approximately 15–17 hours after the HCG injection, the oocytes-cumulus complexes were collected from the oviducts of superovulated female mice. Then, the complexes from several female mice were placed in fertilization medium. Insemination was performed by adding the pre-incubated sperm suspension to the fertilization medium containing complexes and cultured at 37°C with 5%CO2 in air. Twenty-four hours after insemination, 2-cell embryos were transferred into the oviducts of pseudopregnant ICR females (CLEA Japan, Tokyo, Japan) mated to vasectomized ICR males.

### Behavioral analyses

Comprehensive behavioral analyses were performed as described in Table 1 [49]. Male and female mouse pups were used to record USV, induced from maternal separation, although only males were used for all other behavior tests to avoid any influence from hormonal conditions. Four litters were analyzed to confirm the reproducibility.

**Table 1:** Number of animals used in each experiment.

		Young		Aged	
Behavioral tests		WT	*Sey/+*	WT	*Sey/+*
Ultrasonic vocalization	P6	25	24	22	18
Open-field test	8W	12	12	12	10
Social interation test	9-10W	12	11	12	10
Light/dark transition test	11W	12	11	12	9
Home-cage activity	12-13W	12	11	12	10
Fear-conditioning test	14W	12	11	12	10
Tail suspension test	15W	12	11	12	9
Prepuse inhibition test	17W	12	11	12	9

### Measurement of ultrasonic vocalization (USV)

Procedures for maternal separation-induced pup’s USV calls were performed as described in literature [50, 51]. Each pup was recorded on postnatal day 6. The pup was separated from its mother and littermates, one at a time, and placed on a plastic dish with new wood chips made with same material used in home-cage in a soundproof chamber. The room temperature was kept constant at 22~26°C. USV calls were recorded for 5 min with a microphone connected with the UltraSound Gate 416H detector set (Avisoft Bioacoustics, Germany) at 25–125 kHz to measure the number of USV calls and latency until first USV calls.

### Open-field test

Open-field test was conducted at the age of 8 weeks. Each mouse was placed in the corner of an open-field apparatus (40 wide x 40 long x 30 cm high; O’Hara & Co., Ltd., Tokyo, Japan) made of white polyvinyl chloride. The center zone was defined as a square 7.5 cm apart from the wall. The distance traveled, locomotion time, average speed and time in center zone by each animal in the open field were recorded for 20 min with a video-imaging system (Image OF9; O’Hara & Co., Ltd.).

### Three-chamber social interaction test

We performed Crawley’s sociability test as previously described [52], at the age of 9–10 weeks. The apparatus comprised a rectangular, three-chambered box and a lid containing an infrared video camera (O'Hara & Co.). Each chamber was 20 wide × 40 long × 22 cm high and the dividing walls were made from clear Plexiglass, with small square openings (5 × 3 cm) allowing access into each chamber. The subject mouse was placed in the middle chamber and allowed to explore the entire apparatus for a 10 min session one week before the test for habituation. After one week from the habituation, we performed social novelty test. Firstly, habituation session for 10 min was performed again. As for the social interaction test, an unfamiliar male mouse (C57BL/6J) was used for a stranger mouse and was placed in a wire cage in one side chamber. The total time spent near each cage (<4.5 cm) during a 10-min period was determined with a video-imaging system purchased from a commercial supplier (Time CSI2, O’Hara & Co.).

### Light/Dark transition test

A commercially available light/dark chamber (O'Hara & Co., Ltd., Tokyo, Japan) was used for the light/dark transition test at the age of 11 weeks. The apparatus consisted of a light chamber (20 wide × 20 long × 25 cm high) made of white vinyl chloride plates and a dark chamber with the same dimensions made of black vinyl chloride plates. The apparatus had an opening (5 wide × 3 cm high) in the middle of the wall that joins the two chambers. The opening was controlled by a guillotine door. The time spent in the light chamber was measured.

### Home-cage activity

Home-cage activity was examined at the age of 12–13 weeks. Each mouse was placed alone in a testing cage (22.7 wide x 32.9 long x 13.3 cm high) under a 12-h light–dark cycle (light on at 08:00 h) and had free access to both food and water. After one day of acclimation, spontaneous activity in the cage was measured for 5 days (starting at 08:00) with an infrared sensor (activity sensor, O’Hara & Co., Ltd.).

### Tone-fear-conditioned memory test

We performed fear-conditioning test as previously described [53], at the age of 14 weeks. On the training day (day 1), each mouse was placed in a shocking chamber with white wall (O’Hara & Co. Ltd) (Box A) and 120 seconds later, 4 tone-shock pairs were given at 90 seconds intervals. Each tone-shock pair consisted of tone (70db, 10kHz) for a 30 seconds and a foot shock of 2s at 0.5 mA. The foot shock was presented to mice last two seconds of the tone. On day 3, each mouse was put in a white transparent chamber (Box B), and 180s later, 180s tones were delivered. Freezing during the first 180s was “no-tone” in Box B (*i*.*e*., response to an unconditioned context), and freezing in the next 180s was determined as the response to the tone.

### Tail-suspension test

We conducted tail-suspension test in order to examine the depression-like behavior at the age of 15 weeks. The tail of mouse was fixed to the metal plate using adhesive tape. Then the mouse was suspended by the metal plate in the experimental box (40 wide x 40 long x 30 cm high; O'Hara and Co.). The duration of the immobility of the mouse was measured with video analyzing system (O'Hara & Co.) for 6 min.

### Prepulse inhibition test

We performed prepulse inhibition (PPI) test at the age of 17 weeks. Load cell, mouse chamber, sound generator, and sound-proof box (43 wide x 33 long x 33 cm high) were purchased from a commercial supplier (O'Hara & Co.). Before each testing session, mechanical responses were calibrated. Mouse was acclimated to chamber for 5 minutes (only 65 dB background noise was on). During this period, 110 dB/40 ms of white noise was presented to for 5 times in order to acclimate mice to startle pulse. Startle response to these stimuli were excluded from the statistical analysis. Prepulse sounds (75 dB, 80 dB, 85 dB, for 20 ms) and a startle sound (110 dB, for 50 ms) were presented 10 times in pseudorandom order, with an inter-trial interval varying randomly between 10 and 20 seconds and startle amplitude was measured 50 ms after presentation of the prepulse sound. Percentage PPI was calculated as [(startle amplitude without prepulse)–(startle amplitude of trial with prepulse)]/(startle amplitude without prepulse) × 100.

### Statistic analyses

All data are presented as the mean ± standard error of the mean (SEM) and were analyzed using two-way analysis of variation (ANOVA), three-way ANOVA followed by *post hoc* testing Bonferroni and Student *t*-test. SSPS 16.0 software (SPSS Inc., Chicago, USA) was used for statistic analyses and *p*<0.05 was considered statistically significant.

## Results

### *Sey/+* mutant mice show reduced ultrasonic vocalizations

**Fig 1:**
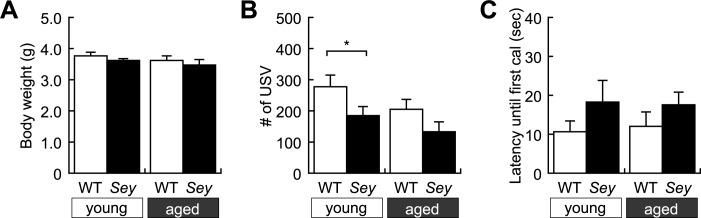
Ultrasonic vocalization of WT and *Sey/+* mouse pups. USV of each mouse pup was recorded during maternal separation on postnatal day 6. Histogram showing results of (A) body weight; two-way ANOVA: no main effect of “genotype”; *F* = 2.269, *p* = 0.136, “father’s age”; *F* = 2.129, *p* = 0.148, and interaction (father’s age x genotype); *F* = 0.024, *p* = 0.876, (B) number of USV calls; two-way ANOVA: main effect of “genotype”; *F* = 6.467, *p* = 0.013, but no main effect of “father’s age”; *F* = 3.465, *p* = 0.066 and interaction (father’s age x genotype); *F* = 0.110, *p* = 0.741, and (C) latency until first USV calls; two-way ANOVA: no main effect of “genotype”; *F* = 2.513, *p* = 0.117, “father’s age”; *F* = 0.005, *p* = 0.944, and interaction (father’s age x genotype); *F* = 0.057, *p* = 0.812, in WT and *Sey/+* mouse pups born to young (3M) or aged (>12M) father. All data are presented by the mean ± SEM. *p < 0.05, versus WT littermate, determined by Bonferroni *post hoc* test. WT: wild type, *Sey*: *Pax6* mutant.

Maternal separation induces USV calls in pups, being one of the earliest behaviors to be tested postnatally. In this experiment, we examined pups’ USV at postnatal day 6 when the number of USV calls reaches the maximum developmental peak [52]. Body weight was comparable among the four groups, i.e., *Sey/+* and WT mice born from young or aged father, suggesting there was no apparent developmental abnormality due to IVF (Fig 1A). The number of USV calls in *Sey/+* pups born from young father was significantly decreased compared to that in WT pups (Fig 1B). In contrast, we observed no significant differences between WT and *Sey/+* pups born from aged father (Fig 1B). No significant difference was detected in both the latency to the first USV call between WT and *Sey/+* pups born from young or aged father (Fig 1C). These results suggest that *Sey/+* pups born from young father exhibit less vocalization when isolated from their mother and littermates; the phenotype was not obvious in *Sey/+* pups born from aged father due to the decreased number of USV calls that occur also in WT littermates. Since USV in pups is considered to be a mother call related to anxiety and perception of temperature [54, 55], *Sey/+* pups born from young father may exhibit a lower level of responsiveness to anxiety or less perception of temperature induced by maternal separation compared with the WT littermates. WT pups born from aged father exhibited a tendency of decrease in USV calls, which was not statistically different from WT pups born from young father. This could be a reason why there was no statistical significance in USV calls between WT and *Sey/+* pups born from aged father.

### Paternal aging induces hyperactivity in *Sey/+* mice and mood abnormalities in WT and *Sey/+* mice

**Fig 2:**
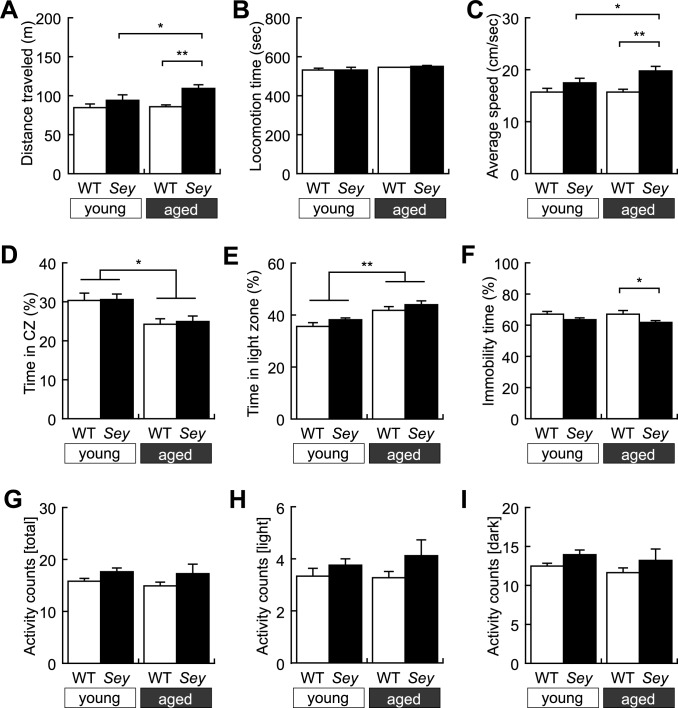
Hyperlocomotor activity and anxiety-like behaviors of WT and *Sey/+* mice. Locomotor activity of each mouse was recorded in the open-field test. Histogram showing results of (A) distance traveled; two-way ANOVA: main effect of “genotype”; *F* = 10.985, *p* = 0.002, but no main effect of “father’s age”; *F* = 2.636, *p* = 0.112, and interaction (father’s age x genotype); *F* = 1.852, *p* = 0.181, (B) locomotion time; two-way ANOVA: no main effect of “genotype”; *F* = 0.054, *p* = 0.818, and “father’s age”; *F* = 3.020, *p* = 0.090, and interaction (father’s age x genotype); *F* = 0.123, *p* = 0.727, (C) locomotion speed; two-way ANOVA: main effect of “genotype”; *F* = 11.017, *p* = 0.002, but no main effect of “father’s age”; *F* = 2.640, *p* = 0.112, and interaction (father’s age x genotype); *F* = 1.852, *p* = 0.181, and (D) time spent in the center zone; two-way ANOVA: main effect of “father’s age”; *F* = 9.432, *p* = 0.004, but no main effect of “genotype”; *F* = 0.003, *p* = 0.959, and interaction (father’s age x genotype); *F* = 0.013, *p* = 0.911 in the open field test. Anxiety-related behavior of each mouse was recorded in the light/dark transition test. Histogram showing results of (E) time spent in the light zone; two-way ANOVA: main effect of “father’s age”; *F* = 17.315, *p* < 0.001, but no main effect of “genotype”; *F* = 2.332, *p* = 0.134 and interaction (father’s age x genotype); *F* = 0.027, *p* = 0.871 in the light/dark transition test. Depression-related behavior of each mouse was recorded in the tail suspension test. Histogram showing results of (F) ratio of immobility time; two-way ANOVA: main effect of “genotype”; *F* = 6.304, *p* = 0.016, but no main effect of “father’s age”; *F* = 0.328, *p* = 0.570, and interaction (father’s age x genotype); *F* = 0.220, *p* = 0.641. Locomotor activity in home cage of each mouse was recorded in the home-cage activity test. Histogram showing results of distance traveled (G) during all the day; two-way ANOVA: no main effect of “genotype”; *F* = 2.579, *p* = 0.116 and “father’s age”; *F* = 0.222, *p* = 0.640, and interaction (father’s age x genotype); *F* = 0.051, *p* = 0.822, (H) during the light period; two-way ANOVA: no main effect of “genotype”; *F* = 2.888, *p* = 0.097 and “father’s age”; *F* = 0.154, *p* = 0.697, and interaction (father’s age x genotype); *F* = 0.359, *p* = 0.552 and (I) during night period; two-way ANOVA: no main effect of “genotype”; *F* = 2.084, *p* = 0.156 and “father’s age”; *F* = 0.551, *p* = 0.462, and interaction (father’s age x genotype); *F* = 0.005, *p* = 0.942 in the home-cage activity test in WT and *Sey/+* mouse pups born to young (3M) or aged (>12M) father. All data are presented by the mean ± SEM. *p < 0.05, ** *p* < 0.01, *** *p* < 0.001, determined by Bonferroni *post hoc* test. WT: wild type, *Sey*: *Pax6* mutant.

Next we performed the open field test to examine locomotor activity and anxiety-related behaviors. No significant difference was observed in distance traveled between WT and *Sey/+* mice born from young father (Fig 2A). Intriguingly, the distance traveled in *Sey/+* mice born from aged father was significantly increased than that in their WT littermates and that in *Sey/+* mice born from young father (Fig 2A). Likewise, locomotion speed during moving, but not locomotion time, was selectively increased in *Sey/+* mice born from aged father compared with WT littermates and *Sey*/+ mice born from young father (Fig 2B and 2C). In contrast, time in the center zone was commonly decreased in both WT and *Sey/+* mice born from aged father than that in both WT and *Sey/+* born from young father (Fig 2D). In another anxiety-related behavior paradigm, the light/dark transition test, both WT and *Sey/+* mice born from aged father spent much time in the light zone than both genotypes born from young father did (Fig 2E), which was superficially an opposite phenotype observed in the open field test (Fig 2D). Thus, *Sey/+* mice born from aged father selectively exhibit hyper locomotion, which could be attributed to increased locomotion speed, but not locomotion time. Although the anxiety-related phenotypes seemed to show discrepancy, paternal aging consistently affected anxiety-related mood statuses in both WT and *Sey/+* mice.

The tail suspension test was conducted to examine the level of depression. Although immobility time was not different between *Sey/+* and WT mice born from young father, *Sey/+* mice born from aged father exhibited decreased time in immobility than in WT littermates (Fig 2F). This result may also relate with the influence of paternal aging on hyperactivity; *Sey/+* mice born to aged father, but not from young father, exhibit hyperactive in the tail suspension test similar to the open field test.

To examine whether the hyperactivity observed in the open-field test and the tail suspension test of *Sey/+* mice born from aged father is specific in a novel environment, we conducted the home cage activity test. No significant difference was observed in total activity counts among the four groups (Fig 2G). To examine light-dark circadian home-cage activity, we evaluated the activity during the light or dark period. No significant difference was observed in activity counts during the light period (Fig 2H) and the dark period (Fig 2I) among the four groups. Taking above findings together, it is assumed that *Sey/+* mice born from aged father were rather hyperactive than their WT littermates; these phenotypes seem to be specific in a novel environment and not attributed to disrupted circadian activity rhythm.

### *Sey/+* mice exhibit no change in social behavior, fear memory formation, and sensorimotor gating

**Fig 3:**
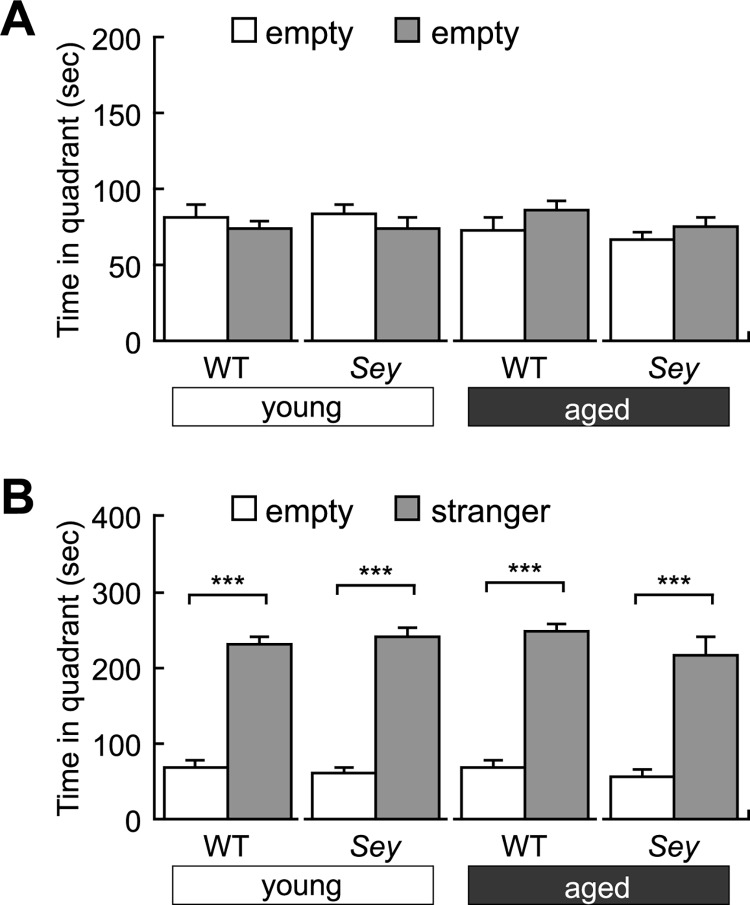
Sociability of WT and *Sey/+* mice. Sociability of each mouse was recorded in the three-chamber social interaction test. Histogram showing results of (A) time spent with two empty compartments and (B) with one stranger compartment in the three-chamber social interaction test using WT and *Sey/+* mouse pups born to young (3M) or aged (>12M) father, No significant difference is found in time spent with two empty compartments in WT and *Sey/+* mice derived from young or aged father (Student *t*-test, *p* > 0.05); in contrast, WT and *Sey/+* mice derived from young or aged father spent more time with stranger compartment than that with empty compartment (Student *t*-test, *p* < 0.001). All data are presented by the mean ± SEM. *** *p* < 0.001, versus WT littermate, determined by Student *t*-test. WT: wild type, *Sey*: *Pax6* mutant.

We conducted the three-chamber test that is commonly used to examine sociability [56] using offspring born from young or aged *Sey/+* father. In our experimental paradigm, all four groups showed no significant difference in stay with two empty compartments (Fig 3A). However, both WT and *Sey/+* mice born from young or aged father spent significantly more time with the stranger mouse than the empty compartment (Fig 3B). Therefore, it is assumed that *Sey/+* mice exhibit comparable sociability to WT littermates regardless of their paternal age.

**Fig 4:**
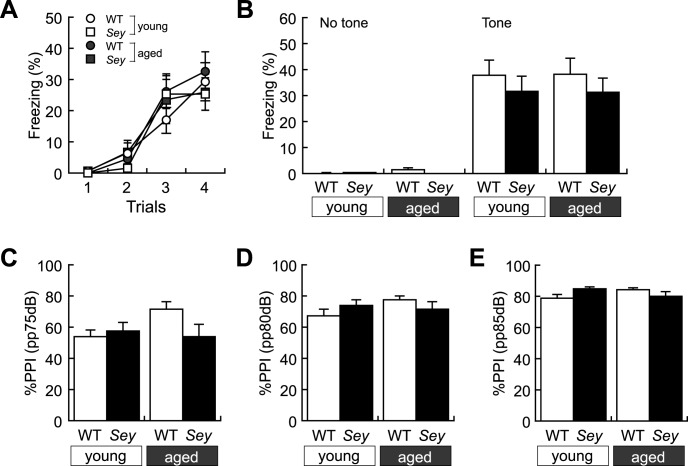
Fear memory and sensorimotor gating of WT and *Sey/+* mice. Fear memory of each mouse was recorded in the cue-dependent fear conditioning test. Histogram showing results of (A) freezing ratio (%) for conditioning; three-way ANOVA: main effect of “trial”; *F* = 34.448, *p* < 0.001, but no main effect of “genotype”; *F* = 0.167, *p* = 0.683, “father’s age”; *F* = 0.599, *p* = 0.440, and interaction (father’s age x genotype); *F* = 0.100, *p* = 0.753, (father’s age x trial); *F* = 0.109, *p* = 0.955, (genotype x trial); *F* = 0.509, *p* = 0.676, (father’s age x genotype x trial); *F* = 0.657, *p* = 0.580, (B) freezing ratio (%); two-way ANOVA: no main effect of “genotype”; *F* = 1.252, *p* = 0.270 and “father’s age”; *F* < 0.001, *p* = 0.985, and interaction (father’s age x genotype); *F* = 0.015, *p* = 0.904 in the cue-dependent fear conditioning test. Sensorimotor gating of each mouse was recorded in the prepulse inhibition test. Histogram showing results of %PPI in (C) prepulse (pp) 75dB; two-way ANOVA: no main effect of “genotype”; *F* = 1.683, *p* = 0.202 and “father’s age”; *F* = 1.892, *p* = 0.177, and interaction (father’s age x genotype); *F* = 3.981, *p* = 0.053, in (D) pp80dB; two-way ANOVA: no main effect of “genotype”; *F* = 0.010, *p* = 0.922 and “father’s age”; *F* = 1.021, *p* = 0.318, and interaction (father’s age x genotype); *F* = 2.500, *p* = 0.122, and in (E) pp85dB; two-way ANOVA: no main effect of “genotype”; *F* = 0.295, *p* = 0.590 and “father’s age”; *F* = 0.057, *p* = 0.813, and interaction (father’s age x genotype); *F* = 5.438, *p* = 0.025 in the prepulse inhibition test in WT and *Sey/+* mouse pups born to young (3M) or aged (>12M) father. All data are presented by the mean ± SEM. *p < 0.05, determined by Bonferroni *post hoc* test. WT: wild type, *Sey*: *Pax6* mutant.

We also conducted the cue-dependent fear-conditioning test to examine fear memory. There was no change in sensitivity against foot shock among the four groups from visual judgment. Moreover, Both WT and *Sey/+* mice born from young or aged father gradually exhibited comparable freezing behavior on the trial in response to presentation of tone and foot shock during the initial training period (Fig 4A). When tested 48 h after the auditory-cue conditioning, both WT and *Sey/+* mice born from young or aged father exhibited similar freezing behavior in response to presentation of the conditioned stimuli (Fig 4B). These results suggest that fear-conditioned memory formation is conserved in both WT and *Sey/+* mice born from young or aged father.

To examine sensorimotor gating functions, we evaluated percentage of PPI to acoustic stimuli (110 dB) with or without prepulse (75, 80, or 85dB). PPI scores were not changed between WT and *Sey/+* mice born to young father and aged father under each condition with 75 (Fig 4C), 80 (Fig 4D) and 85dB (Fig 4E) prepulse, although those were gradually increased in all four groups (Fig 4C–4E). Therefore, *Sey/+* mice born from young or aged father seemed to have comparable sensorimotor gating functions to WT littermate born from young or aged father.

## Discussion

In the present study, we conducted comprehensive behavioral analyses in WT and *Pax6* heterozygous (*Sey/+*) mice born from young or aged *Sey/+* father. Intriguingly, we observed differential effects of father’s age on behavior phenotypes in *Sey/+* mice (Table 2). Less vocalization was selectively observed in *Sey/+* mice born from young father (Fig 1B). In contrast, *Sey/+* mice born from aged father, but not from young father, exclusively showed hyperactivity in the open-field test (Fig 2A and 2C) and in the tail suspension test (Fig 2F). In contrast, both WT and *Sey/+* mice born from aged father exhibited abnormalities in mood statuses compared with those born from young father in the open-field test (Fig 2D) and in the light/dark transition test (Fig 2E). Our results demonstrate, for the first time, that a confounding factor, i.e., paternal age, differential affects behavior phenotypes of mutant mice with a mutation in a neurodevelopmental gene, *Pax6*.

**Table 2:** Summary of behavioral analyses showing differential effect of paternal aging on *Sey/+* behavior phenotypes.

		Young		Aged	
Behavioral tests		WT	*Sey/+*	WT	*Sey/+*
Ultrasonic vocalization	# of USV	-	↓*	-	-
Open-field test	distance traveled	-	-	-	↑*^,#^
	locomotion time	-	-	-	-
	locomotion speed	-	-	-	↑*^,#^
	time in CZ	-	-	↓	↓
Light/dark transition test	time in light zone	-	-	↑	↑
Tail suspension test	immobility time	-	-	-	↓*
Home-cage activity	activity count	-	-	-	-
Social interaction test	time in quadrant	-	-	-	-
Fear-conditioning test	%freezing	-	-	-	-
Prepulse inhibition test	%PPI	-	-	-	-

Among these abnormal behaviors, less vocalization is likely to be caused by *Pax6* haploinsufficiency because we have reported the decreased number of USV in *Pax6* heterozyous rat (*rSey*^*2*^*/+*) [37]. However, the phenotype seems to be masked in *Sey/+* mice born from aged father because their WT littermates showed the lower number of USV calls. Intriguingly, less vocalization seemed to be dominantly observed in male *Sey/+* mice, but not in female, born from young father, although the sample size was not big enough for a definitive conclusion. If this is true, the gender difference may be opposite to that observed in female *rSey*^*2*^*/+* rats [37]. We need to keep our eyes on the gender-dependent phenotypes in our future study.

Immobility in the tail suspension test and anxiety-like behavior in the open-field test, but not in the light/dark transition test, could be explained by paternal aging because a literature reports that immobile time in the forced swim test and time of open-arm in the elevated plus maze are decreased in offspring born to aged father [47]. In contrast, hyper locomotion and increased locomotion speed seem to be more complex and unexplained by a simple influence of *Pax6* haploinsufficiency or paternal aging. Although previous reports revealed that locomotion activity did not change in offspring born from young or aged father [46, 47], paternal aging induced hyper locomotion only in *Sey/+* offspring, but not in WT littermates. Hyperactivity is not reported in *rSey*^*2*^*/+*, in which the father’s age was not considered [37]. In the present study, *Sey/+* mice exhibited no abnormality in social behavior, fear memory formation and sensorimotor gating. In addition, decreased vocalization seemed to be dominantly observed in male pup (now shown here). In our previous analyses, *rSey*^*2*^*/+* rats exhibits abnormal social behavior, i.e. more aggression and less following, in the reciprocal social interaction test, decreased fear memory in the cue-dependent fear conditioning test, and the less number of maternal-separation induced USV calls only in females. The discrepancy in the behavior phenotypes might be attributed to species difference besides the methodological differences. Alternatively, Pax6 protein is undetectable in *rSey*^*2*^*/+* rats, while truncated Pax6 protein is expressed in the developing brain of *Sey/+* mice [57]. If the abnormal Pax6 remains in the brain of *Sey/+* mice, this could function as a dominant active molecule. Another difference is the involvement of IVF in the current study in mice. We do not think that IVF itself may affect offspring’s behavior based on our already accumulated experience in RIKEN BRC. However, we would like to further analyze behavior phenotypes of *Sey/+* mice generated by natural mating.

Taking together, some phenotypes observed in *Sey/+* mice could only be induced in combination of a genetic risk and an environmental risk (e.g., paternal aging). This is a novel aspect that deserves a particular attention in studying animal behaviors; we need to consider father’s age, not only the mother’s, when we conduct behavior analyses to model disease traits.

In this study, we applied IVF to exclude the paternal effect on offspring, since the presence of male mice during gestation can influence fetal development through intrauterine signals such as hormones, immune factors, nutrients and odors [58, 59]. There are several reports pointing out that IVF may relate with abnormal emotional behaviors in adult mouse offspring [60–64]. In this study, *Sey/+* mice born from IVF showed decreased USV vocalizations and less anxiety, which is similar to *rSey*^*2*^*/+* rats born from natural mating [37]. The latter effect could be a consequence of species-specific differences in behavioral phenotypes. We should also continue to keep this potential confounding factor in mind in future studies.

How *Pax6* haploinsufficiency can contribute to behavioral discrepancy in offspring born from young or aged father? It is well known that *Pax6* is expressed in specific spatiotemporal patterns during mammalian brain development and governs neurogenesis and gliogenesis [17–22]. In addition, we recently identified Pax6 expression in male germ line cells, i.e., spermatogonia and spermatocytes, in the testis [65]. Thus, we assume two possible mechanisms for differential behavior phenotypes observed in *Sey/+* mice born from young or aged father; (1) paternal aging may affect spermatogenesis synergistically with paternal *Pax6* haploinsufficiency, thereby epigenetically altering the neurodevelopmental program in their offspring, or (2) paternal aging and *Pax6* dysfunction in offspring may independently affect the offspring’s neurodevelopment. In the second case, *Sey/+* offspring may be more vulnerable than the WT littermates to the influence from paternal aging. Although further studies are necessary to conclude these possible mechanisms, *Pax6* is considered to be the first gene that synergistically functions with paternal aging. Taking mutual expression in the brain and testis, many other genes can also work in a similar way.

At this moment, there are several GWAS studies on ADHD [66–73], none of which unfortunately identified significant association in any gene loci. Regarding this, Brendgem et al. have pointed out a possibility that the phenotype can only be expressed when both genetic and environmental factors are combined [74]. Human *PAX6* gene was originally identified in the chromosome region 11p13 that is responsible for WAGR syndrome [26, 27]. WAGR patients exhibit mental retardation and sometime autistic phenotypes. There are literatures reporting mutations related with *PAX6* gene in patients with ASD, intellectual disabilities, and/or aggressiveness [28–32]. 11p13 region is considered to be related with ASD from an initial GWAS study by The Autism Genome Project Consortium [75, 76]. However, *PAX6* has not been suggested in relation with ASD in more recent genetic studies.

In the present study, we found impaired ultrasonic vocalization in *Sey/+* mice born to young father by maternal separation, i.e., one of the major phenotypes in autism-like behaviors in mice. Our results suggest another possibility of *PAX6* mutation as a vulnerable factor for hyperactivity if it is combined with paternal aging. An intriguing possibility could be that such hyperactivity in mice might be related with aggressiveness in human patients with *PAX6* mutation (see summary of literatures in [28]); it is indeed very difficult to evaluate aggressiveness in mice. In case of *Pax6* mutant rats, in which father’s age was not considered, we did not observe hyperactivity, yet observe some with aggressiveness in social interaction test using the open field [37].

Our study alerts a notion that mice with one single genetic risk factor can develop different phenotypes depending on other confounding factors such as advanced paternal age. On the other hand, our findings imply a limitation in association studies in genetics that frequently include various confounding factors, among which paternal ages are the most difficult to follow. Although neurodevelopmental disorders are thought to be multigenetic diseases, there may be a possibility that diverse symptoms often diagnosed in patients of neurodevelopmental diseases can be attributed to a relatively small numbers of risk genes whose functions are affected by other confounding factors.
